# Quantitative Analysis of Rutin by HPTLC and In Vitro Antioxidant and Antibacterial Activities of Phenolic-Rich Extracts from *Verbesina sphaerocephala*

**DOI:** 10.3390/plants10030475

**Published:** 2021-03-03

**Authors:** Kathia Yanelly Rodríguez-Valdovinos, Rafael Salgado-Garciglia, Monserrat Vázquez-Sánchez, Dioselina Álvarez-Bernal, Ernesto Oregel-Zamudio, Luis Fernando Ceja-Torres, José Roberto Medina-Medrano

**Affiliations:** 1 Centro Interdisciplinario de Investigación para el Desarrollo Integral Regional Unidad Michoacán, Instituto Politécnico Nacional, Jiquilpan 59510, Michoacan, Mexico; yanelly_94@hotmail.com (K.Y.R.-V.); dalvarezb@ipn.mx (D.Á.-B.); eoregel@ipn.mx (E.O.-Z.); lfceja@ipn.mx (L.F.C.-T.); 2 Instituto de Investigaciones Químico-Biológicas, Universidad Michoacana de San Nicolás de Hidalgo, Morelia 58030, Michoacan, Mexico; rsalgado@umich.mx; 3 Programa de Posgrado en Botánica, Colegio de Postgraduados Campus Montecillo, Texcoco 56230, Estado de Mexico, Mexico; vazquez.monserrat@colpos.mx; 4 Programa de Doctorado en Ciencias en Bioprocesos, Unidad Profesional Interdisciplinaria de Biotecnología (UPIBI), Instituto Politécnico Nacional, Ticomán 07340, Ciudad de Mexico, Mexico; 5 CONACYT—Centro Interdisciplinario de Investigación para el Desarrollo Integral Regional Unidad Michoacán, Instituto Politécnico Nacional, Jiquilpan 59510, Michoacan, Mexico

**Keywords:** phenolic compounds, antimicrobial activity, scavenging activity, high-performance thin-layer chromatography (HPTLC), rutin

## Abstract

*Verbesina sphaerocephala* A. Gray, like other wild plants of the genus *Verbesina*, has been used in herbal medicine. There is information for other species of the genus related to their phenolic content, antioxidant capacity, and isolation of bioactive compounds with antimicrobial activity. However, there are no reports for *V. sphaerocephala*, although it has an important presence in the state of Michoacán, México. In this study, the phenolic composition, quantification of rutin, and in vitro antioxidant and antibacterial activities of methanolic extracts from *V. sphaerocephala* leaves and flowers were determined. The results showed that all the investigated extracts have high phenolic and flavonoid contents. The flavonoid rutin was identified in all the extracts from *V. sphaerocephala* by high-performance thin-layer chromatography (HPTLC). The *V. sphaerocephala* extracts showed scavenging activity against DPPH^•^ and ABTS^•+^ radicals (IC_50_ and 5.83 ± 0.50 and 0.93 ± 0.01 mg/mL, respectively) as well as relevant antioxidant capacity (51.05 ± 0.36 mg of ascorbic acid/g of dry tissue). The experimental results show that *V. sphaerocephala* extracts possessed a strong antimicrobial activity against *Escherichia coli* and *Staphylococcus aureus* bacteria. This research indicates that *V. sphaerocephala* could be considered as a potential source of natural compounds from the point of ethnopharmacological usage.

## 1. Introduction

Phenolic compounds, the most abundant secondary metabolites in plants, have received more and more attention in recent years because of their distinct bioactivities [1]. Flavonoids constitute the largest group of phenolic compounds [2]. According to their chemical structure, they are divided into several sub-classes: flavanones, flavanols, flavones, isoflavones, flavonols, and anthocyanins. The flavonoid rutin (quercetin-3-*O*-rhamnoglucoside) is a natural flavonol with a low molecular weight that is widely distributed in plants [3]. Rutin has been reported to have high radical scavenging activity [4], antioxidant capacity [5], and antibacterial activity [6].

The family Asteraceae is considered one of the largest flowering plant families including more than 23,600 species and about 1620 genera [7]. The genus *Verbesina* belongs to the family Asteraceae and contains about 300 species distributed from Canada to Argentina, with the highest diversity in the highlands of Mexico and in the north and central Andes [8]. In Mexico, this genus consists of 179 species [9]. *Verbesina sphaerocephala* A. Gray is an endemic species of Mexico [10] which has been used in traditional medicine for healing, for its anti-inflammatory and antidiarrheal properties, and for the treatment of gangrene, venous ulcer, and gynecological conditions [11]. However, as far as we know, no studies describing the bioactive potential of this plant exist in the literature. 

Therefore, the aim of the present study was to determine the phenolic composition, rutin content, and in vitro antioxidant and antibacterial activities of methanolic extracts from *V. sphaerocephala* leaves and flowers.

## 2. Material and Methods

### 2.1. Chemicals and Reagents

The 2,2-diphenyl-1-picrylhydrazyl (DPPH^•^), 2,2′-azino-bis[3-ethylbenzothiazoline-6-sulphonic acid] (ABTS**^•^^+^**), Folin–Ciocalteu reagent, potassium persulfate, sodium phosphate, sodium carbonate, ammonium molybdate, ascorbic acid, aluminum chloride, ethyl acetate, formic acid, glacial acetic acid, sulfuric acid, methanol, water (HPLC grade), and *p*-anisaldehyde were purchased from Sigma-Aldrich (St. Louis, MO, USA). Plates 20 × 10 cm HPTLC Silica gel 60 F_254_ were purchased from Merck (Kenilworth, NJ, USA). Nutrient agar media and nutrient broth were purchased from BD Bioxon (Estado de México, México). Ampicillin was purchased from AMSA Laboratories (Jalisco, México) and dimethyl sulfoxide (DMSO) was purchased from Fermont (Nuevo León, México).

### 2.2. Standards

Gallic acid, catechin, and rutin (>99%) were purchased from Sigma-Aldrich (St. Louis, MO, USA). The stock solutions were prepared by diluting the standards in methanol (1 mg/mL). The stock solutions were then diluted in methanol to obtain working solutions (0.1–1 mg/mL). Working solutions of standards were stored at −20 °C in dark conditions.

### 2.3. Plant Material 

*V. sphaerocephala* specimens were collected during October 2017 at Pajacuarán, in the Mexican state of Michoacán (20°6′15.001″ N, 102°32′32.42″ W, and 1827 m altitude). Two populations were collected, namely, Population 1 (P1) and Population 2 (P2). The specimens were authenticated by one of the authors, Dr. Monserrat Vázquez Sánchez. Voucher herbarium specimens were deposited at the Jorge Espinoza Salas Herbarium of the Universidad Autónoma Chapingo (UACh), with collection number MVS_62.

### 2.4. Drying Process

Foliar (leaf) and reproductive tissues (flower) were removed from the *V. sphaerocephala* plants and dried in a horizontal air flow oven model TE-FH45DM (Terlab, Mexico) at 40 °C for 24 h. Afterwards, the tissues were ground in a blender to obtain a fine powder. Additionally, to homogenize the particle size to 250 µm, a sieve (number 60) was used. Milled tissues were stored in dark conditions at room temperature until they were used.

### 2.5. Preparation of Extracts

The extraction of phenolic compounds was achieved using 1 g of dry milled plant materials which was suspended in 20 mL of solvent (50% methanol, 80% methanol, or 100% methanol, *v/v*) by agitation at 100 rpm using an orbital shaker apparatus, model Sea Star (Heathrow Scientific, Vernon Hills, IL, USA), in the dark at room temperature for 24 h. Then, the extracts were centrifuged at 2722× *g* for 10 min at room temperature. The supernatant was recovered and filtered through Whatman No. 1 filter paper (Whatman International Ltd., Maidstone, UK) to obtain the crude extract. Aliquots of the extract were taken for the phenolic content, chromatographic analysis, and antioxidant determinations.

### 2.6. Determination of Total Phenolic and Flavonoid Contents

#### 2.6.1. Total Phenolic Content

The determination of total phenolic contents of the extracts was conducted using the Folin–Ciocalteu method with some modifications [12]. The extracts (250 µL) were mixed with 1250 µL of distilled water, followed by 62.5 µL of 1N Folin–Ciocalteu reagent, and stirred for 5 min. Lastly, 187.5 µL of 20% (*w/v*) Na_2_CO_3_ solution was added and kept up in dark conditions for 2 h at room temperature. The absorbance of each sample was read at 760 nm using a Microplate Spectrophotometer (PowerWave HT, BioTek Instruments, Inc., Winooski, VT, USA). Total phenolic contents were estimated using a gallic acid standard curve (A_760_ = 0.0023 [gallic acid] + 0.0355, *R*^2^ = 0.9943), obtained using eight known concentrations (40–460 µg/mL) of gallic acid. The total phenolic content was expressed as milligrams of gallic acid equivalents per gram of dry tissue (mg GAE/g DT).

#### 2.6.2. Total Flavonoid Content

Total flavonoid content of each sample was determined by the aluminum chloride method previously reported, with slight modifications [13]. Five hundred microliters of extract was added with 500 µL of 2% (*w/v*) solution of AlCl_3_·6H_2_O. After 10 min, absorbance was read at 430 nm using a PowerWave HT Microplate Spectrophotometer (BioTek Instruments, Inc., Winooski, VT, USA). Total flavonoid contents were estimated using a catechin standard curve (A_430_ = 0.0121 [catechin] − 0.0032, *R*^2^ = 0.9906) obtained using eleven concentrations of catechin (400–4250 µg/mL). Total flavonoid contents were expressed as milligrams of catechin equivalents per gram of dry tissue (mg CE/g DT).

### 2.7. Quantitative Analysis and Radical Scavenging Activity of Rutin by High-Performance Thin-Layer Chromatography (HPTLC)

A Camag HPTLC instrumental set-up (Camag, Muttenz, Switzerland) consisting of an Automatic Sample Applicator (ATS 4), an Automatic Developing Chamber (ADC 2), a Chromatogram Immersion Device III, a TLC Plate Heater III, a TLC Visualizer, and VisionCats version 2.5.18072.1 data processing software was used for the analysis. Standard and sample solutions (5 μL) were applied bandwise (band length 8 mm, 15 nL/s delivery speed under nitrogen 6 bars pressure, track distance 9.4 mm, and distance from the edge 20.1 mm) on a HPTLC glass plate (20 × 10 cm) coated with 200 μm layer thickness of silica gel 60 F_254_ by an Automatic TLC Sampler (ATS 4). Plates were developed in an Automatic Developing Chamber (ADC 2) with ethyl acetate/formic acid/acetic acid/water (100:11:11:26, *v/v/v/v*) as the mobile phase (70 mm). The saturation time of the chamber was conditioned and optimized to 5 min at room temperature (22 °C ± 2) and relative humidity (33 ± 2%) for better resolution with mobile phase vapors. The plates were dipped in 200 mL of freshly prepared *p*-anisaldehyde-sulfuric/acetic acids solution (170 mL of methanol, 20 mL of glacial acetic acid, 5 mL concentrated sulfuric acid, and 1 mL of *p*-anisaldehyde), using a Chromatogram Immersion Device IIP (speed 50 mm/s, time 1 s), and subsequently heated (100 °C, 10 min) in a TLC Plate Heater III. The chromatograms were documented under visible and UV (λ_max_ 254 and 366 nm) light after development and after post-chromatographic derivatization with the use of a TLC Visualizer 2 and a computer program, VisionCats version 2.5.18072.1. 

For screening of the antioxidant activity of the separated chromatographic zones, the developed plate was dipped for 1 s in freshly prepared 0.1% (*w/v*) DPPH^•^ radical solution using a Chromatogram Immersion Device III and subsequently heated at 40 °C (2 min) on a TLC Plate Heater III. Documentation of the chromatograms was performed at white light illumination.

Rutin was identified in the derivatized plates (with *p*-anisaldehyde-sulfuric/acetic acids solution) based on the band colors as well as retardation factors (R_f_) of the standard in comparison with constituents of the analyzed extracts. For quantitative analysis, the content of rutin was determined by using a calibration curve established with a standard concentration range from 0.1 to 1 mg/mL per spot. The quantification of rutin was accomplished based on the regression equation (*y* = [4.484 × 10^−1^ *x* / 2.472 × 10^−7^ + *x*] + 5.67 × 10^−1^, *R* = 99.9872%) of each concentration peak area plotted against the concentration of rutin spotted (0.1, 0.3, 0.5, 0.7, 1.0, and 1.2 mg/mL), and the results were expressed as mg/g of dry tissue (mg/g DT).

### 2.8. Antioxidant Activity Assays

#### 2.8.1. DPPH^•^ Antioxidant Assay

The determination of free radical scavenging activity was determined according to the DPPH^•^ method previously described [14]. Firstly, a 24 µM ethanol solution of DPPH^•^ was prepared. Then, 450 µL of DPPH^•^ reagent previously prepared was mixed with 50 µL of extract (1.25–25 mg/mL) and they were incubated for 10 min at room temperature. After incubation, the absorbance was measured at 523 nm using a Microplate Spectrophotometer (PowerWave HT, BioTek Instruments, Inc., Winooski, VT, USA). 

The scavenging effect of DPPH^•^ was measured using the formula

(1) DPPH• scavenging effect (%) = [(Acontrol − Asample ) / Acontrol] × 100

where *A*_control_ is the absorbance of the control (DPPH^•^ solution), and *A*_sample_ is the absorbance of the test sample (DPPH^•^ solution plus 50 µL of extract). The median inhibitory concentration (IC_50_) was determined using linear regression. The scavenging activity was expressed as the IC_50_ that represents the *V. sphaerocephala* extract concentration (mg/mL) needed to reduce by 50% the initial DPPH^•^ absorbance. 

#### 2.8.2. ABTS^•+^ Antioxidant Assay

The ABTS^•+^ radical scavenging assay was used to evaluate the antioxidant activity of the extracts [15]. For this purpose, a 7 mM ABTS^•+^ solution (dissolved in distilled water) was prepared. ABTS^•+^ radical cations were produced by reacting 1 mL of ABTS^•+^ stock solution with 17.6 µL of 140 mM potassium persulfate. The mixture was kept up in the dark at room temperature for 12 h before use. After, the ABTS^•+^ solution was diluted with deionized water to obtain an absorbance of 0.70 (±0.01) at 734 nm. Finally, 500 µL of diluted ABTS^•+^ radical solution was added to 500 µL of the extract (0.01–2.5 mg/mL, dissolved in their respective extraction solvents). After 6 min, absorbance was registered at 734 nm using a PowerWave HT Microplate Spectrophotometer (BioTek Instruments, Inc., Winooski, VT, USA). 

The scavenging effect percentage was calculated using the formula

(2) ABTS•+ scavenging effect (%) = [(Ablank − Asample ) / Ablank] × 100

where *A*_blank_ represents the absorbance of the blank (ABTS^•+^ solution plus 500 µL of 50% methanol, 80% methanol, or 100% methanol (*v/v*), and *A*_sample_ is the absorbance of the *V. sphaerocephala* extracts (ABTS^•+^ solution plus 500 µL of extract). The IC_50_ (mg/mL) was calculated by using linear regression.

#### 2.8.3. Total Antioxidant Capacity Assay

The phosphomolybdenum assay was used to evaluate the total antioxidant capacity [16]. In this method, the formation of a green phosphate/Mo (V) complex for the reduction of Mo (VI) to Mo (V) by an antioxidant is measured at an acidic pH. To accomplish this, 50 µL of *V. sphaerocephala* extract was combined with 500 µL of a solution that contained 0.6 M sulfuric acid, 28 mM sodium phosphate, and 4 mM ammonium molybdate. Then, extracts were incubated in a digital dry bath (Thermo Fisher Scientific, Inc., Waltham, MA, USA) at 95 °C for 90 min. After incubation, the samples were subsequently cooled down to room temperature, and the absorbance of each was measured at 695 nm using a Microplate Spectrophotometer (PowerWave HT, BioTek Instruments, Inc., Winooski, VT, USA). A blank formed by 50 µL of 50% methanol, 80% methanol, or 100% methanol (*v/v*) instead of extract was used. The total antioxidant capacity was calculated by using an ascorbic acid standard curve (A_695_ = 0.0014 [ascorbic acid] − 0.011, *R*^2^ = 0.9993) generated with six concentrations of ascorbic acid (0.03–0.3 mg/mL). Total antioxidant capacity was expressed as milligrams of ascorbic acid equivalents per gram of dry tissue (AAE/g DT).

### 2.9. Antibacterial Analysis

#### 2.9.1. Sample Preparation for Antibacterial Analysis

Prior to antibacterial analysis, the extracts (20 mL) were placed on a Petri plate in a fume hood at ambient temperature for 24 h to allow evaporation of the solvent. Subsequently, each extract was transferred to a 15mL Falcon tube, and 5 mL of distilled water was added. Then, the extracts were lyophilized using a Labconco freeze dryer, model 77530 (Labconco, Kansas City, MO, USA), at −50 °C for 24 h and under high vacuum conditions (0.02 mBar). After this, 20 mg of each extract was resuspended individually in 1 mL of methanol/DMSO (9:1, *v/v*). Finally, the extracts were stored under refrigeration at 4 °C protected from light.

#### 2.9.2. Antibacterial Activity

The antibacterial activity of *V. sphaerocephala* extracts was surveyed by employing the disc diffusion assay to determine the inhibition zones of all samples against *Escherichia coli* strain 0111 and *Staphylococcus aureus* separately, provided by Instituto de Diagnóstico y Referencia Epidemiológicos (InDRE), Ministry of Health, Mexico City. Each strain was grown in nutrient broth at 37 °C. After 24 h, each microorganism at 1 × 10^6^ CFU/mL was inoculated on the surface of nutrient agar media plates. A sterile inoculation loop was used to streak-inoculate each fresh nutrient agar media plate. Twenty-five microliters of the extracts (25 mg/mL) were loaded on sterile blank discs (5 mm diameter) and the discs were impregnated onto inoculated agar. Discs with ampicillin (10 mg/mL, dissolved in DMSO) were used as positive controls and discs impregnated with methanol/DMSO (9:1, *v/v*) were used as negative controls. The plates were incubated at 25 °C ± 2 for 24 h. The diameters of the inhibition zones (mm) indicating the antibacterial activity of the extracts were manually measured with a manual vernier caliper (Stanley Black & Decker, Inc., New Britain, CT, USA). Each inhibition zone diameter was measured three times (three different plates).

The values of bacteria growth inhibition during the treatment with extracts used (after 24 h) was calculated according to the formula

(3) Growth inhibition (%) = (Dsample − Dcontrol ) × 100

where *D*_sample_ represents the inhibition zone diameter of bacteria samples treated with the extracts (disc with 25 μL of the extracts), and *D*_control_ is the inhibition zone diameter of the control sample (discs with ampicillin).

### 2.10. Statistical Analysis

Results were reported as mean ± standard deviation of three independent replicates. An analysis of variance (ANOVA) was used to assess statistical significance. Differences between values with a *p* < 0.05 were considered statistically significant. For the comparison of means for the corresponding results, Tukey’s test was performed. For the antibacterial activity assays, Dunnett’s test was used to assess the statistical significance of differences between test and control data (*p* < 0.05). Relationships between all determinations were tested using Pearson’s correlation. These analyses were performed with the SPSS software version 25.0 (SPSS Inc., Chicago, IL, USA).

## 3. Results and Discussion

### 3.1. Total Phenolic and Flavonoid Contents

In this work, our aim was to estimate the total phenolic and flavonoid contents of the methanolic extracts from *V. sphaerocephala* leaves and flowers, and the results are presented in Table 1. The total phenolic contents of the *V. sphaerocephala* extracts ranged from 6.55 ± 0.71 to 10.50 ± 0.76 mg GAE/g DT. The highest phenolic content was observed in leaves from Population 2 extracted with 100% methanol (P2-L-100M), and the lowest was observed in leaves from Population 1 extracted with 100% methanol (P1-L-100M). 

In the case of flower tissue, the highest concentration of phenolic compounds was found in Population 2 extracted with 100% methanol (P2-F-100M), while Population 1 extracted with 50% methanol (P1-F-50M) showed the lowest phenolic content. The concentration of total phenolics in the methanolic extracts from *V. sphaerocephala* Population 2 (both tissues) was higher than Population 1 according to the statistical analysis (*p* < 0.05). The mean total phenolic content value of the extracts of *V. sphaerocephala* was 8.45 mg GAE/g DT. Previous research results have shown a concentration of 3.23 mg GAE/g DT in ethanolic extracts of *V. sphaerocephala* leaves [17], lower values compared to those obtained from the extracts analyzed here.

**Figure 1:**
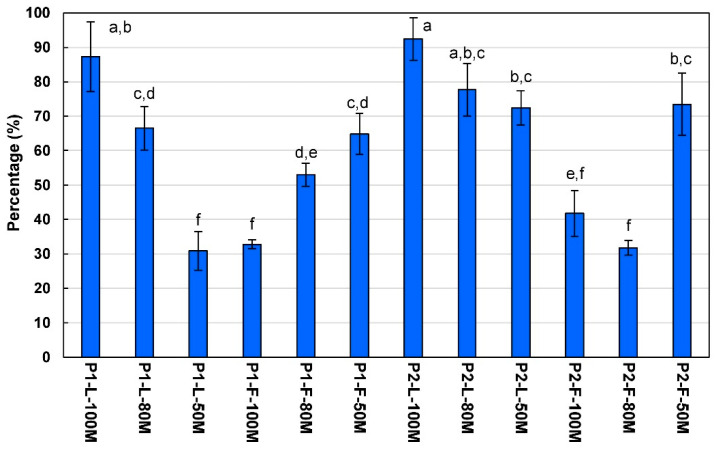
Average percentage of total flavonoid content from the total phenolic content of methanolic extracts from *Verbesina sphaerocephala* leaves and flowers. Bar plot represents mean ± standard deviation of three repetitions. Different letters indicate significant differences between groups within homogeneous subsets (Tukey, *p* < 0.05). Abbreviations: P1, Population 1; P2, Population 2; L, leaves; F, flowers; 100M, extracted with 100% methanol; 80M, extracted with 80% methanol; 50M, extracted with 50% methanol.

Regarding the total flavonoid content, as seen in Table 1, the lowest content was observed in leaf extracts from Population 1 extracted with 50% methanol (P1-L-50M), with 2.09 ± 0.35 mg CE/g DT, while the highest content was observed in leaf extracts from Population 2 extracted with 100% methanol (P2-L-100M), with 9.85 ± 0.38 mg CE/g DT. The previous extract also showed the highest percentage of flavonoids (92.4%) with respect to the total phenolic content, which is shown in Figure 1.

In the case of flower extracts, the lowest value of the total flavonoid content was shown by Population 2 extracted with 80% methanol (P2-F-80M), while the highest flavonoid content was obtained in Population 2 extracted with 50% methanol (P2-F-50M). The above values represent 31.7 and 73.5%, respectively, of the total phenolic content (Figure 1). The concentration of the total flavonoid content in the methanolic extracts from *V. sphaerocephala* leaves and flowers was lower compared to previously published data (37.83 mg CE/g DT) [17].

Based on Pearson’s correlation coefficient, no clear relationship was observed between the total phenolic and flavonoid contents of the extracts (*R* = 0.601, *p* < 0.01) since the extracts that showed the highest total phenolic contents did not obtain the highest total flavonoid contents.

### 3.2. Quantitative Analysis and Radical Scavenging Activity of Rutin by HPTLC

**Figure 2:**
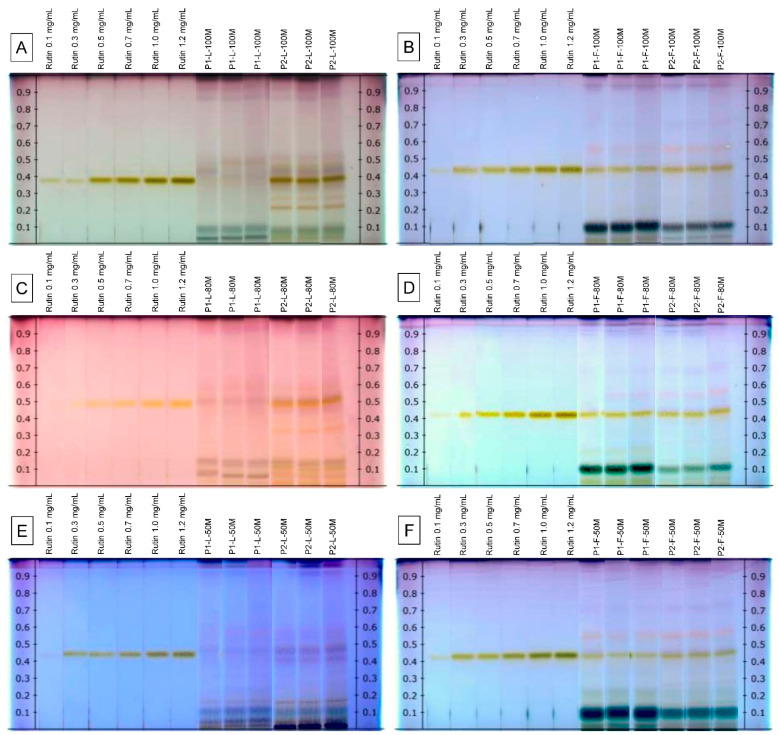
HPTLC chromatogram of methanolic extracts from *Verbesina sphaerocephala* under visible light in the transmittance mode after derivatization with *p*-anisaldehyde. (**A**) Leaf tissue from Populations 1 and 2 extracted with 100% methanol. (**B**) Flower tissue from Populations 1 and 2 extracted with 100% methanol. (**C**) Leaf tissue from Populations 1 and 2 extracted with 80% methanol. (**D**) Flower tissue from Populations 1 and 2 extracted with 80% methanol. (**E**) Leaf tissue from Populations 1 and 2 extracted with 50% methanol. (**F**) Flower tissue from Populations 1 and 2 extracted with 50% methanol.

Derivatization with *p*-anisaldehyde/sulfuric acid reagent was used to detect rutin in the *V. sphaerocephala* extracts. The *p*-anisaldehyde-sulfuric acid reagent, under visible light, reacts with phenolic compounds to form differently colored zones. The analysis by HPTLC showed clearly separated compact, sharp, and high-resolution, yellow-colored bands in the plates derivatized with the *p*-anisaldehyde-sulfuric acid reagent. Rutin bands (yellow) were observed in all the extracts from *V. sphaerocephala* analyzed in this study. The bands of rutin were obtained at R_f_ 0.380–0.439 (Figure 2). It is noteworthy that the *V. sphaerocephala* flower profiles showed great similarity in the rutin signals, while in the leaves, the profiles showed notable differences.

HPTLC fingerprint analysis of *V. sphaerocephala* extracts was, for the first time, performed in the present work. As observed in Table 1, the highest content of rutin was found in the extract P2-L-80M, extracted from leaves of Population 2 with 80% methanol (13.97 ± 4.09 mg/g DT), which was about 11 times higher than the extract with the lowest content (P1-L-100M, 1.21 ± 0.22 mg/g). Overall, extracts from Population 2 showed a rutin content superior to the rest (*p* < 0.05). The concentrations of rutin of the 100%, 80%, and 50% methanolic extracts from *V. sphaerocephala* leaves and flowers are presented in Table 1. The rutin content in the extracts from *V. sphaerocephala* showed a tendency, since the highest rutin contents were observed in samples that obtained the highest total phenolic and flavonoid contents. According to the analysis, a positive correlation was observed between the total phenolic and flavonoid contents and the rutin content (*R* = 0.689, *p* < 0.01 and *R* = 0.563, *p* < 0.01, respectively) of the *V. sphaerocephala* extracts. 

To the best of our knowledge, the presence of rutin has not been previously reported for the *V. sphaerocephala* species analyzed in this work. Previous chemical investigations of *V. sphaerocephala* species have shown the presence of phenolic compounds such as bornyl ferulate, bornyl *p*-coumarate, tyrosol, icariside D_2_, and hyperin [18]. Identification of rutin in extracts should be helpful for further analysis.

According to Figure 2, bands of various colors (pinks, violets, blues, and grays) were observed in all extracts, suggesting the presence of different classes of natural products in the *V. sphaerocephala* extracts; thus, the authors recommend carrying out a phytochemical screening of the plant for future research.

**Figure 3:**
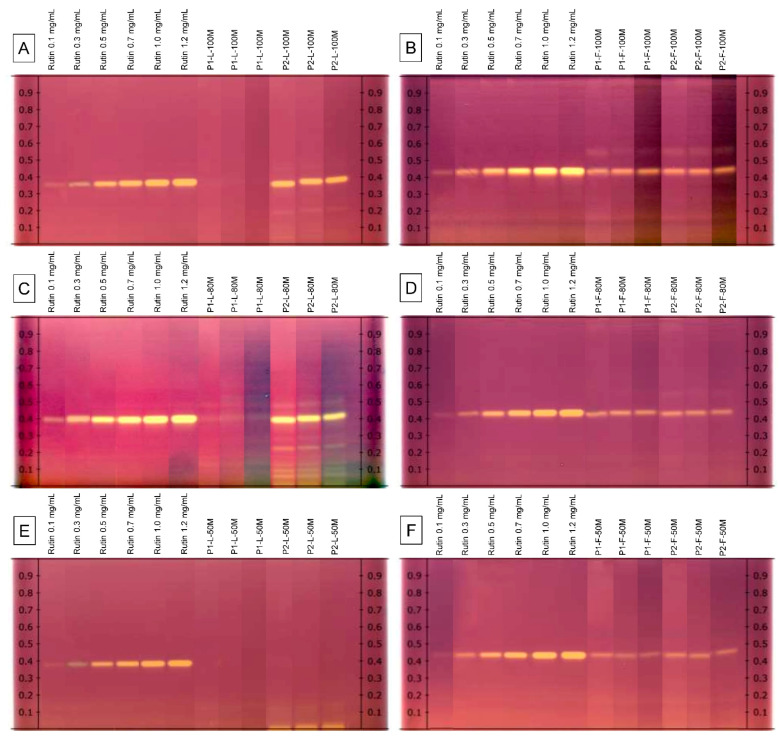
HPTLC chromatogram of methanolic extracts from *Verbesina sphaerocephala* under visible light in the transmittance mode after derivatization with DPPH^•^ radical solution. (**A**) Leaf tissue from Populations 1 and 2 extracted with 100% methanol. (**B**) Flower tissue from Populations 1 and 2 extracted with 100% methanol. (**C**) Leaf tissue from Populations 1 and 2 extracted with 80% methanol. (**D**) Flower tissue from Populations 1 and 2 extracted with 80% methanol. (**E**) Leaf tissue from Populations 1 and 2 extracted with 50% methanol. (**F**) Flower tissue from Populations 1 and 2 extracted with 50% methanol.

The antioxidative potential of the *V. sphaerocephala* extracts was preliminary investigated by the HPTLC-DPPH^•^ method. This technique allows rapid, on-the-spot evaluation of the capacity of plant extracts to produce a biological effect [19]. The DPPH^•^ free radical molecule is stable at room temperature and need not be generated. This strongly colored free radical is reduced (scavenged) in the presence of an antioxidant molecule from purple to a colorless/yellow reduced form [20]. Therefore, after derivatization with the DPPH^•^ radical, inhibition areas (bright yellow bands) were observed in the bands where rutin was identified (Figure 3). The intensity of inhibition was higher in leaf samples from Population 2 extracted with 100% methanol. The results of the HPTLC–DPPH^•^ test indicate that the rutin bands in *V. sphaerocephala* extracts had a significant effect on the scavenging activity of radical DPPH^•^. 

### 3.3. Antioxidant Activity

To the authors’ best knowledge, there are no previous reports on the antioxidant activity of *V. sphaerocephala* extracts. In this work, *V. sphaerocephala* extracts showed in vitro scavenging activity of DPPH^•^ and ABTS^•+^ radicals, as well as relevant antioxidant capacity. As can be seen in Table 1, a different antioxidant behavior of the extracts was observed. 

According to Table 1, the extract P2-L-100M, extracted from leaves of Population 2 with 100% methanol, exhibited the strongest DPPH^•^ radical scavenging activity with an IC_50_ value of 5.83 ± 0.50 mg/mL. Interestingly, the P2-L-100M extract showed the highest phenolic and flavonoid contents and a high rutin concentration (Figure 1 and Table 1). On the other hand, the leaf extracts from Population 1 (P1-L-100M, P1-L-80M, and P1-L-50M) failed to even reduce by 50% the initial DPPH^•^ concentration; therefore, they were considered as those with the lowest scavenging activity of radical DPPH^•^. In accordance with the above, these extracts showed the lowest total phenolic content (P1-L-100M) and the lowest flavonoid content (P1-L-50M), as well as low rutin contents. Previous studies reported an IC_50_ of 432.56 ± 15.72 μg/mL in methanolic extracts from aerial parts of *Verbesina crocata* [21].

Regarding the scavenging activity of the ABTS^•+^ radical, the most effective scavenging against ABTS^•+^ was obtained by the extract P2-L-50M, with an IC_50_ value of 0.93 ± 0.01 mg/mL. As mentioned earlier, the extract P2-L-50M showed high phenolic and flavonoid contents, as well as moderate concentrations of rutin (Table 1). The concentration required to inhibit the ABTS^•+^ radical shown by the extracts of *V. sphaerocephala* was similar (and in some cases lower) to that reported for *V. croata* [21]. While the methanolic extracts of *V. sphaerocephala* inhibited the absorbance of DPPH^•^ and ABTS^•+^ radicals depending on the extract concentration and showed a dose-dependent tendency, the correlation analysis revealed lower associations between the scavenging activities and the phenolic, flavonoid, and rutin contents in the extracts (*R* = −0.421, *p* < 0.05 between total phenolic content and ABTS^•+^ > *R* = −0.497, *p* < 0.01 between total flavonoid content and ABTS^•+^ > *R* = −0.498, *p* < 0.01 between rutin content and ABTS^•+^). 

The total antioxidant capacity analysis of the extracts from leaves and flowers of two populations of *V. sphaerocephala* ranged from 23.57 ± 0.14 to 51.05 ± 0.36 mg AAE/g DT (Table 1). Similarly, as with the ABTS^•+^ radical, the leaf extract from Population 2, extracted with 50% methanol (P2-L-50M), exhibited the highest antioxidant capacity, while the extracts of flowers from Population 2, extracted with 80% and 100% methanol (P2-F-80M and P2-F-100M), exhibited the lowest antioxidant capacity, with 14.67 ± 0.30 and 15.00 ± 0.29 mg AAE/g DT, respectively. The correlation analysis indicates a significant positive correlation between total antioxidant capacity and the total flavonoid content (*R* = 0.709, *p* < 0.01), which indicates that by increasing the content of flavonoids in the extracts, the total antioxidant capacity increased. There are no previous studies of total antioxidant capacity in *Verbesina* genus extracts with which to compare the antioxidant capacity obtained by extracts of *V. sphaerocephala* analyzed in this study.

### 3.4. Antibacterial Activity

**Figure 4:**
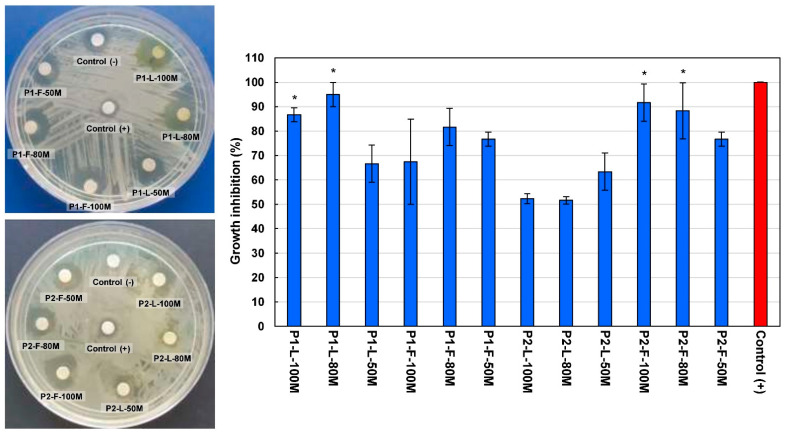
Antibacterial activity of methanolic extracts from *Verbesina sphaerocephala* leaves and flowers against *Escherichia coli*. Values are expressed as mean ± standard deviation of three repetitions. Abbreviations: P1, Population 1; P2, Population 2; L, leaves; F, flowers; 100M, extracted with 100% methanol; 80M, extracted with 80% methanol; 50M, extracted with 50% methanol; Control (+), ampicillin 10 mg/mL; Control (-), methanol/DMSO. (*) Indicates no significant statistical difference from the control according to Dunnett’s test (*p* < 0.05).

**Figure 5:**
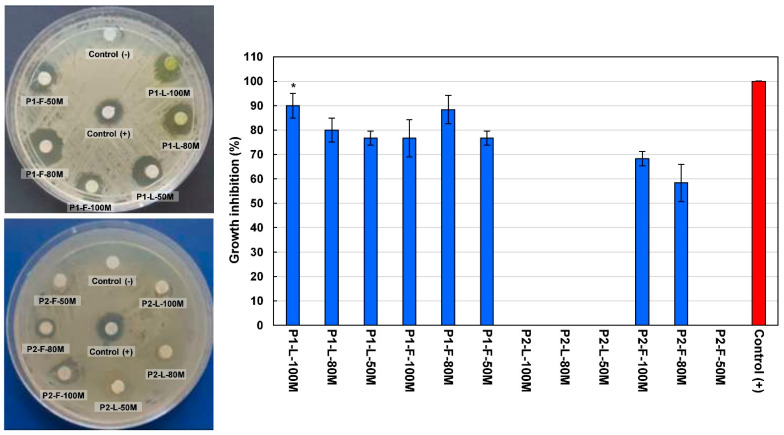
Antibacterial activity of methanolic extracts from *Verbesina sphaerocephala* leaves and flowers against *Staphylococcus aureus*. Values are expressed as mean ± standard deviation of three repetitions. Abbreviations: P1, Population 1; P2, Population 2; L, leaves; F, flowers; 100M, extracted with 100% methanol; 80M, extracted with 80% methanol; 50M, extracted with 50% methanol; Control (+), ampicillin 10 mg/mL; Control (-), methanol/DMSO. (*) Indicates no significant statistical difference from the control according to Dunnett’s test (*p* < 0.05).

The antibacterial activity of methanolic extracts from *V. sphaerocephala* leaves and flowers was screened against *E. coli* and *S. aureus*, and the results obtained are summarized in Figure 4 and Figure 5.

All extracts showed growth inhibition against *E. coli*, ranging from 51.67 to 95.00%. As can be observed in Figure 4, the best results of bacterial growth inhibition against *E. coli* were obtained by the leaf extract from Population 1 extracted with 80% methanol (P1-L-80M), reaching 95.00 ± 5.00% (19 mm) > the flower extract from Population 2 extracted with 100% methanol (P2-F-100M), with 91.67 ± 7.64% (18 mm) > the flower extract from Population 2 extracted with 80% methanol (P2-F-80M), with 88.33 ± 11.50% (17.6 mm) > the leaf extract from Population 1 extracted with 100% methanol (P1-L-100M), with 86.67 ± 2.89% (17 mm). According to Dunnett’s test, no significant difference was found in the bacterial growth inhibition of the extracts mentioned above and the control used (ampicillin, 10 mg/mL).

On the other hand, the best results against *S. aureus* were observed by the methanolic extract from Population 1 (P1-L-100M), with 90.00 ± 5.00% (18 mm), which was not statistically different from the control according to Dunnett’s test (*p* < 0.01). Leaf extracts from Population 2 (P2-L-100M, P2-L-80M, and P2-L-50M), as well as extracts from flowers extracted with 50% methanol (P2-F-50M), were inactive against the *S. aureus* strain at the concentration assayed (Figure 5). Other species of the genus *Verbesina* with reported antibacterial activity are *V. encelioides* and *V. macrophylla* [22,23].

Despite the above, it was not possible to attribute the antibacterial effect shown by the *V. sphaerocephala* extracts to the phenolic compounds determined, since the correlation analysis showed negative correlations between the antimicrobial activity and the total phenolic content (*R* = 0.704, *p* < 0.01), total flavonoid content (*R* = 0.738, *p* < 0.01), and rutin content (*R* = 0.631, *p* < 0.01), which means that, as the content of these compounds decreased, the antibacterial activity increased. Based on the above reason, it can be inferred that the antibacterial activity observed in bioassays could be due to a possible synergistic effect between the phenolic compounds present in the *V. sphaerocephala* extracts.

According to the literature research conducted, there are no reports on the antibacterial activity of *V. sphaerocephala*; thus, the authors recommend broadening the spectrum of bacteria for future investigations.

## 4. Conclusions

To the best of our knowledge, this is the first report of the phenolic composition, separation and simultaneous quantification of rutin, and in vitro antioxidant and antibacterial activities of the different aerial parts of *V. sphaerocephala* extracts. The results of the present study revealed that *V. sphaerocephala* was rich in phenolic compounds. From the results of HPTLC analysis, the presence of rutin was observed in all the methanolic extracts from *V. sphaerocephala* leaves and flowers. Furthermore, experimental results showed that *V. sphaerocephala* methanolic extracts possessed a notable antioxidant power and a strong activity toward the test bacteria. They also indicate that *V. sphaerocephala* may be considered as a potential source of natural antioxidants from the point of ethnopharmacological usage of this plant.

## Figures and Tables

**Table 1:** Total phenolic, flavonoid, and rutin contents, free radical scavenging activity, total antioxidant capacity, and antibacterial activity of methanolic extracts from leaves and flowers of *Verbesina sphaerocephala*.

Sample	TPC (mg GAE/g DT)	TFC (mg CE/g DT)	Rutin (mg/g DT)	DPPH^•^	ABTS^•+^	TAC (mg AAE/g DT)
				IC_50_ (mg/mL)	IC_50_ (mg/mL)	
P1-L-100M	6.55 ± 0.71 ^f^	5.67 ± 0.00 ^c^	1.21 ± 0.22 ^c^	ND	1.99 ± 0.02 ^j^	23. 57 ± 0.14 ^g^
P1-L-80M	7.88 ± 0.45 ^c,d,e,f^	5.23 ± 0.38 ^c,d^	3.07 ± 0.44 ^c^	ND	1.67 ± 0.01 ^g^	22.29 ± 0.14 ^h^
P1-L-50M	6.78 ± 0.20 ^e,f^	2.09 ± 0.35 ^g^	2.53 ± 0.77 ^c^	ND	1.36 ± 0.01 ^d^	30.00 ± 0.14 ^e^
P1-F-100M	8.91 ± 0.31 ^b,c,d^	2.92 ± 0.10 ^f,g^	3.62 ± 0.26 ^c^	12.13 ± 0.10 ^f^	1.89 ± 0.02 ^i^	24.48 ± 0.22 ^f^
P1-F-80M	7.53 ± 0.47 ^d,e,f^	3.99 ± 0.13 ^e^	4.10 ± 0.49 ^c^	11.17 ± 0.12 ^d,e^	1.51 ± 0.01 ^e^	19.19 ± 0.50 ^i^
P1-F-50M	7.18 ± 0.28 ^e,f^	4.64 ± 0.25 ^d,e^	2.57 ± 0.26 ^c^	10.56 ± 0.11 ^d^	1.36 ± 0.01 ^d^	29.29 ± 0.43 ^e^
P2-L-100M	10.50 ± 0.76 ^a^	9.85 ± 0.38 ^a^	7.93 ± 0.48 ^b^	5.83 ± 0.50 ^a^	1.34 ± 0.01 ^d^	44.33 ± 0.22 ^b^
P2-L-80M	10.46 ± 0.86 ^a,b^	8.09 ± 0.38 ^b^	13.97 ± 4.09 ^a^	6.26 ± 0.02 ^a,b^	1.05 ± 0.01 ^b^	34.29 ± 0.29 ^d^
P2-L-50M	10.17 ± 0.35 ^a,b^	7.36 ± 0.25 ^b^	4.87 ± 1.06 ^b,c^	13.66 ± 0.04 ^g^	0.93 ± 0.01 ^a^	51.05 ± 0.36 ^a^
P2-F-100M	9.28 ± 0.69 ^a,b,c^	3.84 ± 0.34 ^e,f^	4.20 ± 0.53 ^b,c^	11.71 ± 0.11 ^e,f^	1.79 ± 0.01 ^h^	15.00 ± 0.29 ^j^
P2-F-80M	8.17 ± 0.43 ^c,d,e^	2.59 ± 0.22 ^g^	4.44 ± 0.50 ^b,c^	9.49 ± 0.25 ^c^	1.60 ± 0.02 ^f^	14.67 ± 0.30 ^j^
P2-F-50M	7.94 ± 0.26 ^c,d,e,f^	5.82 ± 0.55 ^c^	3.76 ± 0.20 ^c^	6.72 ± 0.46 ^b^	1.13 ± 0.00 ^c^	41.29 ± 0.14 ^c^

Abbreviations: TPC, total phenolic content; TFC, total flavonoid content; VS, *Verbesina sphaerocephala*; GAE, gallic acid equivalents; CE, catechin equivalents; AAE, ascorbic acid equivalents; DT, dry tissue; DPPH, 2,2-diphenyl-1-picrylhydrazyl; ABTS, 2,2-azino-bis[3-ethylbenzothiazoline-6-sulphonic acid]; IC_50_, median inhibitory concentration; P1, Population 1; P2, Population 2; L, leaves; F, flowers; 100M, extracted with 100% methanol; 80M, extracted with 80% methanol; 50M, extracted with 50% methanol; ND, not determined. Values represent mean ± standard deviation of three repetitions. Different letters indicate significant differences according to Tukey’s test (*p* < 0.05).

## Data Availability

The authors declare that the data supporting the findings of this study are available within the article.
